# Micro/Nano Periodic Surface Structures and Performance of Stainless Steel Machined Using Femtosecond Lasers

**DOI:** 10.3390/mi13060976

**Published:** 2022-06-20

**Authors:** Xiaofeng Xu, Laifei Cheng, Xiaojiao Zhao, Jing Wang, Xinyi Chen

**Affiliations:** 1 CNPC Tubular Goods Research Institute, Xi’an 710077, China; chenxy1@cnpc.com.cn; 2 Science and Technology on Thermostructural Composite Materials Laboratory, School of Materials Science and Engineering, Northwestern Polytechnical University, Xi’an 710072, China; chenglf@nwpu.edu.cn (L.C.); wangjing1@nwpu.edu.cn (J.W.); 3 School of Electronic Engineering, Xi’an Shiyou University, Xi’an 710065, China; zhaoxiaojiao@xsyu.edu.cn

**Keywords:** femtosecond laser, micro/nano periodic surface structure, surface performance, stainless steel, research progress

## Abstract

The machining of micro/nano periodic surface structures using a femtosecond laser has been an academic frontier and hotspot in recent years. With an ultrahigh laser fluence and an ultrashort pulse duration, femtosecond laser machining shows unique advantages in material processing. It can process almost any material and can greatly improve the processing accuracy with a minimum machining size and heat-affected zone. Meanwhile, it can fabricate a variety of micro/nano periodic surface structures and then change a material’s surface performance dramatically, such as the material’s wetting performance, corrosive properties, friction properties, and optical properties, demonstrating great application potential in defense, medical, high-end manufacturing, and many other fields. In recent years, the research is gradually deepening from the basic theory to optimization design, intelligent control, and application technology. Nowadays, while focusing on metal structure materials, especially on stainless steel, research institutions in the field of micro and nano manufacturing have conducted systematic and in-depth experimental research using different experimental environments and laser-processing parameters. They have prepared various surface structures with different morphologies and periods with sound performance, and are one step closer to many civilian engineering applications. This paper reviews the study of micro/nano periodic surface structures and the performance of stainless steel machined using a femtosecond laser, obtains the general evolution law of surface structure and performance with the femtosecond laser parameters, points out several key technical challenges for future study, and provides a useful reference for the engineering research and application of femtosecond laser micro/nano processing technology.

## 1. Introduction

In recent years, emerging technologies represented by functional micro/nanostructures have become an important direction of the global development of science and technology due to their different material and functional characteristics at the micro/nano scale. With advanced micro/nano processing technology, the biomimetic nature of a micro/nano hierarchical structure on a metal surface can be realized [1,2], forming micro/nano protrusions with different scales, similar to the surface of the lotus leaf. Through the modification of a material with a low surface energy, special wetting metal surfaces with hydrophobic or even superhydrophobic and superoleophobic characteristics can be obtained.

Among many micro/nano processing technologies, such as photolithography, X-ray, electron beam, particle beam, and mechanical methods, femtosecond laser machining shows unique advantages in material processing; owing to its ultrahigh laser fluence and ultrashort pulse duration, the physical and chemical mechanism of the laser–material interaction is fundamentally changed [3,4]. A femtosecond laser can process almost any material, and at the same time can greatly reduce the heat-affected zone, microcracks, and recast layer, coupled with the fact that the nonlinear multiphoton effect during femtosecond laser processing makes the minimum machining size far less than the laser wavelength, and thus can break the optical diffraction limit, which significantly improves the processing accuracy [5,6,7]. Since a femtosecond laser’s pulse duration is usually shorter than or equal to the characteristic time of most physical/chemical processes, a series of interesting new phenomena can be induced by controlling the local transient electronic dynamics during processing. When a material surface is irradiated by a femtosecond laser, a variety of unusual periodic surface structures can be formed, including the most representative surface ripples and quasiperiodic conical spikes, as well as regular arrays of nanorods [8]. Since M. Birnbaum used a laser to obtain a periodic ripple structure on a semiconductor surface in 1965 [9], it has been found that many materials (such as metals, semiconductors, dielectric materials, etc.) will form a periodic surface structure under the induction of a laser [10,11,12,13]. A femtosecond-laser-machined micro/nano periodic surface structure can have a significant impact on the material’s surface performance; change the material’s wetting performance [14,15,16], friction properties [17,18,19], and optical properties [20,21,22]; and prepare a hydrophobic material [23], a bionic structure surface [24], or a colored metal [25], demonstrating a huge application potential in national defense, medical, high-end manufacturing, and many other fields. For example, it can effectively improve the surface corrosion resistance of oil production equipment and tubular goods, realize the self-cleaning effect of a metal surface, and reduce the friction resistance of fluid and a pipeline’s inner wall [26,27], which is expected to result in the upgrading of oil tubular goods and equipment, as well as new technologies for the sustainable development of the oil and gas industry [28].

The machining of micro/nano periodic surface structures using a femtosecond laser has been an international academic frontier and hotspot in recent years. The research is gradually deepening from basic theory to optimal design, intelligent control, and application technology [3,28]. M. D. Shirk et al. [29] presented a review of ultrashort pulsed laser ablation of materials in which they discussed the means of ultrashort pulsed laser generation and some specific examples of ultrashort pulsed machining of metals, polymers, and ceramics, and introduced the laser–material interactions. J. Cheng et al. [30] reviewed the development of ultrafast laser micromachining of materials, and mainly presented general experimental observations, mathematical models and the physics behind the process. As the research focus moved from understanding the complex ablation mechanism to the fabrication and application of surface structures, K. M. Tanvir Ahmmed et al. [3] reviewed the state-of-the-art knowledge on the fabrication of micro/nanostructures on metals with direct femtosecond laser micromachining. Z. Zhu et al. [31] introduced the recent research progress in femtosecond laser micro/nano fabrication for bioinspired superhydrophobic or underwater superolephobic surfaces. Nowadays, while focusing on metal structure materials, especially stainless steel, researchers have carried out extensive research and have gathered a large body of useful data, and are one step closer to realizing many civilian engineering applications. This paper reviews micro/nano periodic surface structures and the performance of the stainless steel material machined using a femtosecond laser, obtains the basic evolution law of micro/nano periodic surface structures with femtosecond laser parameters, and points out several research challenges to be studied, which may provide a useful reference for promoting the engineering research and application of femtosecond laser micro/nano processing technology.

## 2. Preparation of Micro/Nano Periodic Surface Structures

The shape, size, and period of the micro/nano periodic structures produced by femtosecond laser ablation on a material’s surface are not only closely related to the properties of the material itself, but also depend on the experimental research environment and the laser parameters used. Using different experimental environments and laser-processing parameters, worldwide research institutions in the field of micro and nano manufacturing have conducted systematic and in-depth experimental research, and have prepared various surface structures with different morphologies and periods.

### 2.1. Machines, Equipment, and Type of Laser

**Figure 1:**
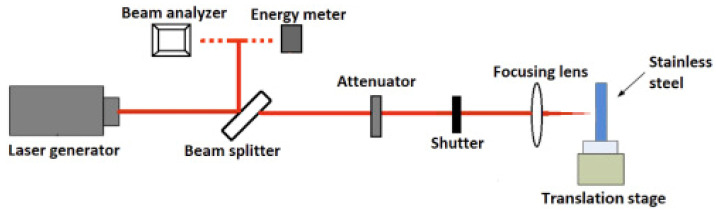
Schematic of laser treatment experimental setup. “Reprinted with permission from Ref. [32], Copyright (2017), Elsevier”.

Normally, a commercial Ti:sapphire chirped-pulse amplification laser system [32,33] is adopted to create a femtosecond laser for the fabrication of a micro/nano periodic surface structure; the schematic layout of such an experimental system is shown in Figure 1. The attenuator is used to adjust the laser energy, the quarter or half wave-length plate is used to control the laser polarization, and the electronic shutter is used to control the number of pulses. Linearly polarized light is used in most studies, and the femtosecond laser pulses are focused on the polished specimen surface using a focusing lens. The target specimen is mounted on a computer-controlled multiaxis motion stage with a high resolution.

**Figure 2:**
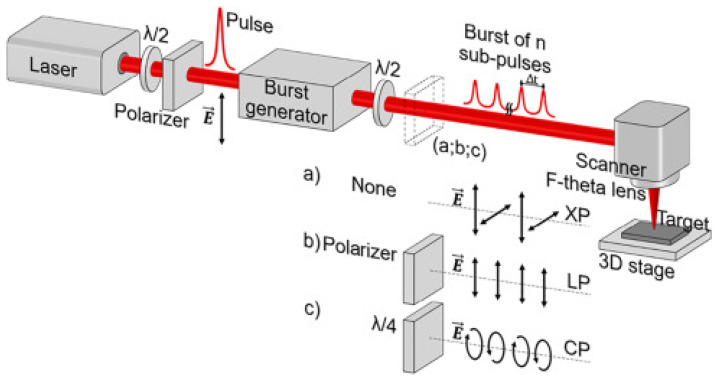
Sketch of the experimental setup used for the generation of the bursts and for the irradiation of the stainless-steel samples. (**a**) The burst of sub-pulses were crossed-polarized (XP); (**b**) The XP bursts were converted into linearly polarized (LP); (**c**) The XP bursts were converted into circularly polarized (CP). “Reprinted with permission from Ref. [34], Copyright (2019), Elsevier”.

Interestingly, Giannuzzi G. et al. [34] employed a new type of laser system that could produce bursts of femtosecond laser pulses, as shown in Figure 2. By means of an array of five calcite birefringent crystals, the pulse was split into *n* subpulses (with *n* from 2 to 32) with equally fractioned energy. The intraburst time delay could be adjusted from 1.5 ps to 24 ps. Typically, the subpulses emerging from the crystal array were alternately crossed-polarized, and they could be converted into a linearly polarized or circularly polarized burst through a polarizer or a quarter-wave plate.

### 2.2. Micro/Nanoripple Structure

Ripple structures consist of two categories: nanoscale laser-induced periodic surface structures (LIPSS) and microscale groove structures. According to the period size, the LIPPS structure is further subdivided into a low-spatial-frequency LIPSS (LSFL), which has a periodicity less than the incident laser wavelength but greater than the half wavelength, and a high-spatial-frequency LIPSS (HSFL), which has a periodicity smaller than the half wavelength.

**Figure 3:**
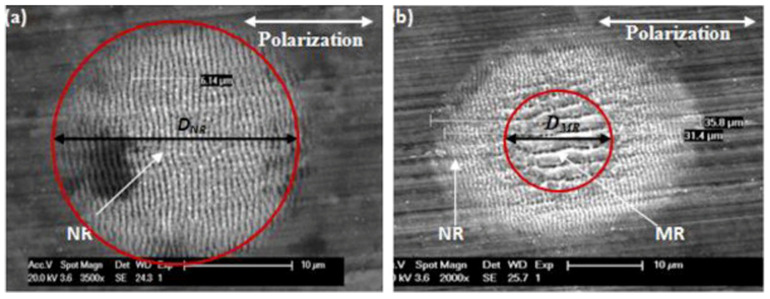
Nano- and microripples on X40Cr14. (**a**) Nanoscale ripples (NR); (**b**) microscale (MR) and nanoscale ripples (NR). “Reprinted with permission from Ref. [36], Copyright (2013), Elsevier”.

**Figure 4:**
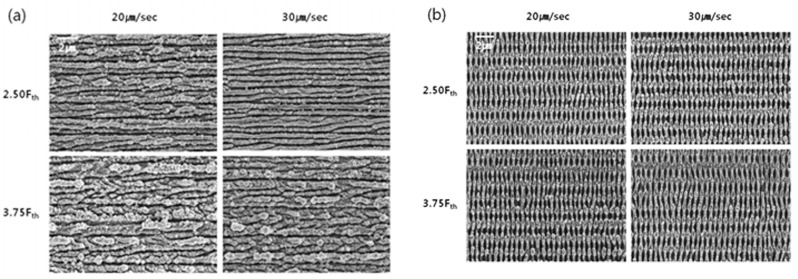
Large range of ripples using multiple line scanning with different scan directions. (**a**) The scan direction is perpendicular to the polarization direction; (**b**) The scan direction is parallel to the polarization direction. “Reprinted with permission from Ref. [37], Copyright (2012), Springer Nature”.

By using a femtosecond laser, Litao Qi et al. [35] prepared regularly arranged LSFL structures and HSFL structures on a stainless steel surface. P. Bizi-bandoki et al. [36] obtained a nanoscale LSFL structure and a microscale ripple structure on a stainless steel surface, as shown in Figure 3. Sang-Hoon Choi et al. [37] used a femtosecond laser for spatially confined micromachining on the mold stainless steel STAVAX to prepare a large range of ripple structures using multiple line scanning, as shown in Figure 4.

**Figure 5:**
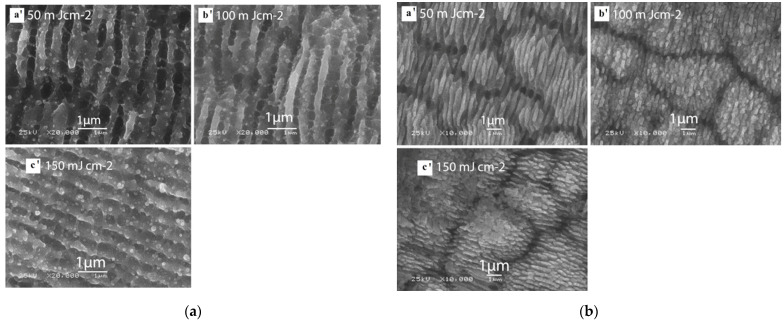
Ripple processing in air environment on AISI 304 stainless steel. (**a**) Center of ablation area with different fluence, (**a’**) 50 mJ cm^−2^; (**b’**) 100 mJ cm^−2^; (**c’**) 150 mJ cm^−2^; (**b**) periphery of ablation area with different fluence, (**a’**) 50 mJ cm^−2^; (**b’**) 100 mJ cm^−2^; (**c’**) 150 mJ cm^−2^. “Reprinted with permission from Ref. [38], Copyright (2013), Springer Nature”.

Shazia Bashir et al. [38] studied the preparation of micro/nano periodic surface structures of stainless steel induced by a femtosecond laser in different environments of air, deionized water, and ethanol, and obtained the ripple structure. In the air environment, as shown in Figure 5, the ripple structure is not continuous, and microscale craters and nanoparticles can be observed. In the periphery of the ablation area, it can be clearly seen that the ripple structure is divided into small islands by continuous nanopores. The ripple structure is smaller and more diffused and dispersed, and the appearance of dots is more distinct in the water environment, while high-density ripples and nano-dots are clearly visible at the center of the ablation region in the ethanol environment.

Micro/nanostructures are always prepared on the surface of a structural material with suitable processing parameters [39,40,41,42,43]. Through special designs and processes, micro/nanoripple structures also are often found together with other structures constituting more complex structural features [32,44,45].

Many factors affect the micro/nano surface structure, mainly including the physical and chemical properties of the material itself; the laser physical parameters related to the femtosecond laser system, such as the laser’s wavelength, pulse width, repetition frequency, fluence, and polarization direction; and the machining parameters, such as the processing environment, scanning speed, radiation direction, and focus position. In view of the ripple structure of alloy structural materials, the researchers carried out related studies focusing on some influencing factors of laser fluence, pulse number, scanning speed, bursts of pulses, and processing environment.

#### 2.2.1. Ablation Threshold and Laser Fluence

It was found that when processing with a femtosecond laser, this special surface ripple structure can be formed only when the laser fluence is above the ablation threshold of the material. This threshold is not only related to the characteristics of the material itself, but also to the processing parameters.

Oskar Armbruster et al. [46] verified that the ablation threshold decreased and stabilized with the laser beam size and decreased with the number of pulses. Shizhen Xu et al. [47] studied the influence of the pulse width (35 fs and 260 fs) and processing environment on the ablation threshold of 304 stainless steel, and found that both an air environment and a shorter pulse width were conducive to reducing the ablation threshold.

Qi L. et al. [35] confirmed that LIPSS structures could be prepared when the laser fluence was in the range of 0.42 to 1.32 J/cm^2^. Compared with the LSFL structure, the ablation threshold of the HSFL structure was lower. When the laser fluence was higher, due to the characteristics of the Gaussian distribution of the femtosecond laser energy, an LSFL structure was obtained in the center of the ablation region while an HSFL structure was obtained in the peripheral region. When the laser fluence was lower, the HSFL structure could be obtained in the entire area. Compared to that of a nanoscale ripple structure, the ablation threshold of a micro ripple structure was higher. Bizi-bandoki P. et al. [36] verified that the mean ablation threshold was 1.51 J/cm^2^ for a microscale ripple structure and 0.10 J/cm^2^ for an LSFL structure, and also found that only the microscale ripple structure period first increased and then decreases with an increasing laser fluence, while the nano ripple structure period was not sensitive to laser fluence changes.

#### 2.2.2. Pulse Number/Scan Number

With a certain laser fluence, an LIPSS structure will evolve along with the number of pulses. Qi L. et al. [35] found that at a laser fluence of 1.32 J/cm^2^, nanoprotrusions with few nanocavities were observed at 5 pulses, nanoscale periodic ripples began to appear at 7, an LSFL structuring of the entire region was obtained at 20 pulses, and the LSFL structure in the middle region began to disappear at more than 100 pulses. At a laser fluence of 0.67 J/cm^2^, the surface structure evolved from LSFL to HSFL, and then to a disordered structure as the number of pulses increased. Bizi-bandoki P. et al. [36] found that nanoscale LSFL structures were sensitive only to the number of pulses, with a 15% reduction in the structure period as the number of pulses increased. Yao C., Xu S. and Yasumaru N. [32,47,48] obtained similar influence laws, with the ripple period decreasing with the number of pulses or the number of scans.

Shuangshuang Hou et al. [49] used a femtosecond laser to produce LSFL ripples and HSFL ripple structures on stainless steel surfaces, and found that the division of LSPL was the decisive factor in the formation of HSFL structures. Bin Liu et al. [50] studied the evolution of a picosecond-laser-induced nano ripple structure on a stainless steel surface and reached a similar conclusion. By maintaining a certain laser fluence, the transition from LSFL ripples to HSFL ripples could be realized by changing the number of pulses.

When the total irradiation energy (laser fluence × pulse number) was certain, the ripples obtained by multiple scans were more regular and the average period was smaller, but the range of the period change was wider, and the surface structure was rougher. The self-organization theory could explain the experimental results well.

#### 2.2.3. Scan Speed

Choi S. H. et al. [37] used a femtosecond laser for spatially constrained micro processing on mold stainless steel STAVAX to investigate the effects of different scanning speeds, different energy densities, and two laser polarization directions on femtosecond-laser-induced linear structures. The single-pulse ablation threshold (*F_th_*) was approximately 80 m J/cm^2^, and the scanning mode ablation threshold was roughly the same as the single-pulse irradiation (0.75 *F_th_*~1.25 *F_th_*). However, the laser fluence needed to reach 2.5 times the *F_th_* during high-speed scanning to obtain a continuous linear structure, and 1.25 times the *F_th_* for low-speed scanning. The width of the laser machining line increased with an increasing laser fluence and decreased with an increasing scanning speed, independent of the polarization direction.

Razi S. et al. [41] found that the period of an LSFL structure increased with an increasing scan speed.

#### 2.2.4. Pulse Bursts

Xingsheng Wang et al. [51] studied the periodic structures induced by linearly polarized multiburst picosecond laser pulses on stainless steel, and also prepared periodic structures of different scales and shapes such as LSFLs, HSFLs, and microgrooves. Giuseppe Giannuzzi et al. [52] studied the effect of the time separation between subpulses and the number of subpulses in the burst on the surface structure of stainless steel. The results showed that when the number of subpulses was two, the period of the ripple structure increased and the depth decreased when the time separation increased; when the time separation was fixed to 1.5 ps, the depth was basically unchanged when the number of subpulses increased.

#### 2.2.5. Experimental Environment

Xu S. et al. investigated the effect of the pulse width and environment on the ablation rate of 304 stainless steel [47]. At a lower laser fluence (less than 0.92 J/cm^2^), the ablation rate was similar despite the different pulse width and experimental environment. At a higher laser fluence (greater than 0.92 J/cm^2^), the ablation rate of a 35 fs pulse width in a vacuum was significantly higher than that of the others. It was considered that the two phases were dominated by the optical penetration depth and the electron diffusion length, respectively [53]. Cui Z. et al. [40] investigated the effect of different environments on the ablation rate of femtosecond-laser-ablated stainless steel. Under single-raster scanning, the ablation rate in a liquid was higher due to the confinement of the plasma, laser-induced shockwaves, and bubble-related recoil forces; under multiple-raster scans, the ablation rate was higher in air due to the preferential ablation of the ablated surface morphology. J.S. Yahng et al. [54] studied the effect of a stainless steel’s matrix temperature on the rate of femtosecond laser ablation. With a substrate temperature ranging from 300K to 900K, the efficiency of femtosecond laser ablation of the stainless steel increased by 20%, while the surface roughness decreased significantly.

Table 1 provides a summary of main test parameters and typical structures of ripples on stainless steels. Most research used 800 nm laser wavelength, however, F. Fraggelakis et al. [43] demonstrated the feasibility of inducing ripples and spikes utilizing a 257 nm femtosecond laser. Besides, some experimental findings used 10 ps are also listed in the table for comparison.

### 2.3. Other Structures

**Figure 6:**
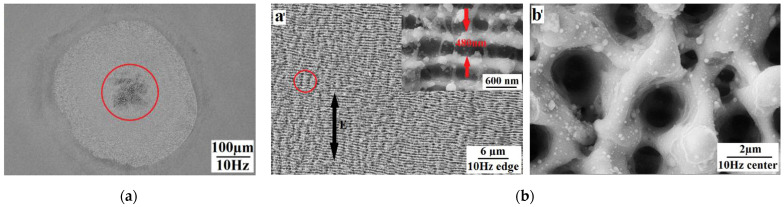
Moth-eye-like structure on 304 stainless steel with repetition rate of 10 Hz. (**a**) Whole ablated area; (**b**) detailed view of micro/nanostructures in peripheral (**a’**) and core region (**b’**). “Reprinted with permission from Ref. [32], Copyright (2017), Elsevier”.

Yao C. et al. [32] prepared a micro/nanostructure on a stainless steel surface using a femtosecond laser with different repetition frequencies (4 Hz, 10 Hz, 500 Hz, 1000 Hz) and different pulses (90, 180, 300), as shown in Figure 6. The microstructure consisted of two distinct regions: the central region of a moth-eye-like structure and the peripheral region of a micro/nano periodic structure surrounding the center. The size of the moth-eye structure increased dramatically with the number of pulses, and the regularity of the micro/nanostructures decreased with an increasing repetition frequency. The authors believed that the microscale periodic ripple structure parallel to the polarization direction was the transition organization from the nanoscale periodic ripple structure to the moth-eye-like structure. The formation of micro/nanostructures could be explained by the plasmon resonance absorption model [57,58].

**Figure 7:**
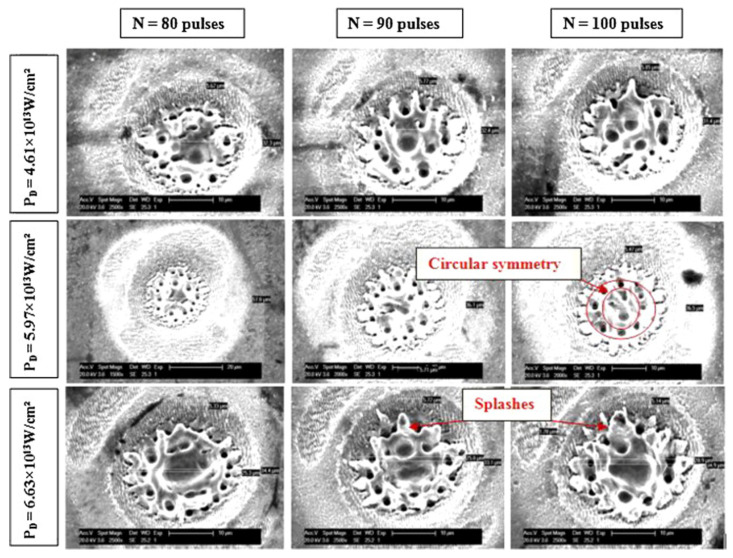
Porous and splashlike structures on X40Cr14. “Reprinted with permission from Ref. [36], Copyright (2013), Elsevier”.

Bizi-bandoki P. et al. [36] prepared porous and splashlike structures in the central ablation region at a higher laser fluence (more than 3.31 J/cm^2^) and number of pulses (more than 70), and the microscale holes were arranged in an orderly circle, as shown in Figure 7. Similar structures were obtained by Maharjan N. and Xu S. [39,47].

**Figure 8:**
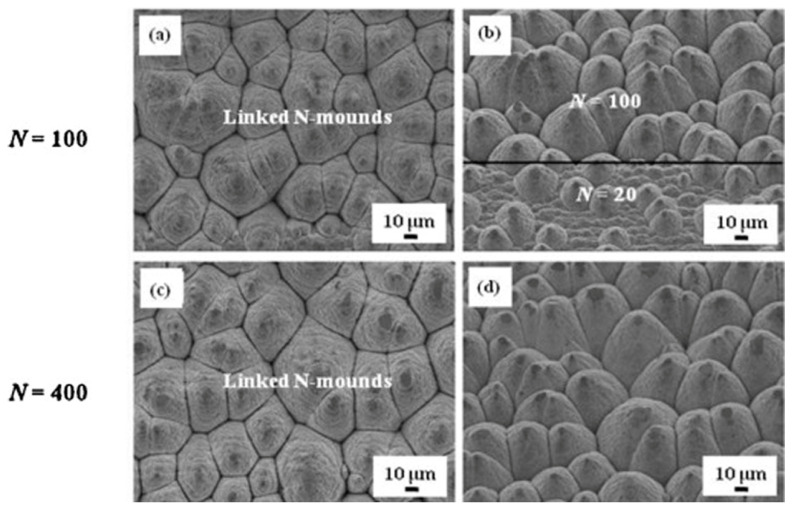
Linked *N*-mound microstructure. (**a**,**c**) were normal to the surface; (**b**,**d**) were taken at a 40° angle to the surface. “Reprinted with permission from Ref. [59], Copyright (2015), Elsevier”.

Li Y. et al. obtained a closely linked *N*-mound structure [59], as shown in Figure 8, and the formation steps and mechanism of the structure were studied in detail. When the mound structure was formed, the surface morphology no longer changed significantly with the number of raster scans. In addition, due to the redeposition of ablated materials, the surface of the mound was covered with concentric ripples and irregular nanostructures. Cui Z. et al. [40] prepared closely linked *N*-mounds and ripple-textured microprotrusions using multiscanning in air and methanol environments, respectively. In the air environment, a submicro ripple structure was formed after the first raster scan. As the number of scans increased, the closely connected mound/cone structure formed after more than 100 scans. In the methanol environment, a ripple structure was also seen first, but it was uneven and covered with nanoparticles measuring 200~300 nm. With an increase in the raster scans, vertical ripple grooves appeared, dividing the ripple structure into more discontinuous short segments that slowly grew and combined, finally forming a ripple-textured microprotrusion structure.

**Figure 9:**
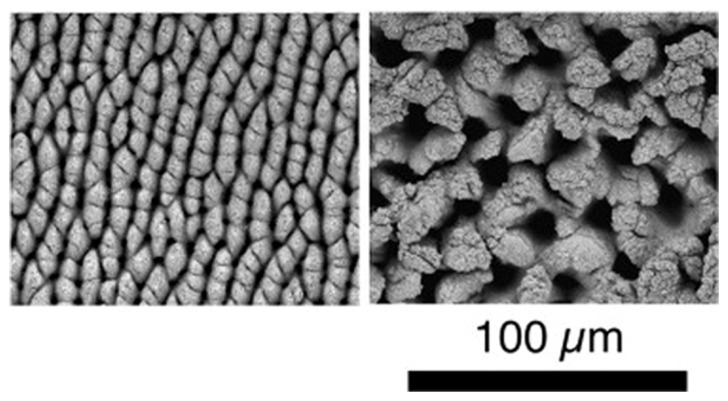
Columnar and chaotic structures on 304 stainless steel. “Reprinted with permission from Ref. [60], Copyright (2015), Elsevier”.

E.J.Y. Ling et al. [60] scanned the surface of 304 stainless steel multiple times with femtosecond laser pulses to obtain an ellipsoidal cone structure at a low laser fluence (cumulative line laser fluence [61] <130 J/cm^2^) and columnar and chaotic structures at a high laser fluence (cumulative line laser fluence >130 J/cm^2^), as shown in Figure 9; such structures were relatively stable and did not change with the number of scans.

**Figure 10:**
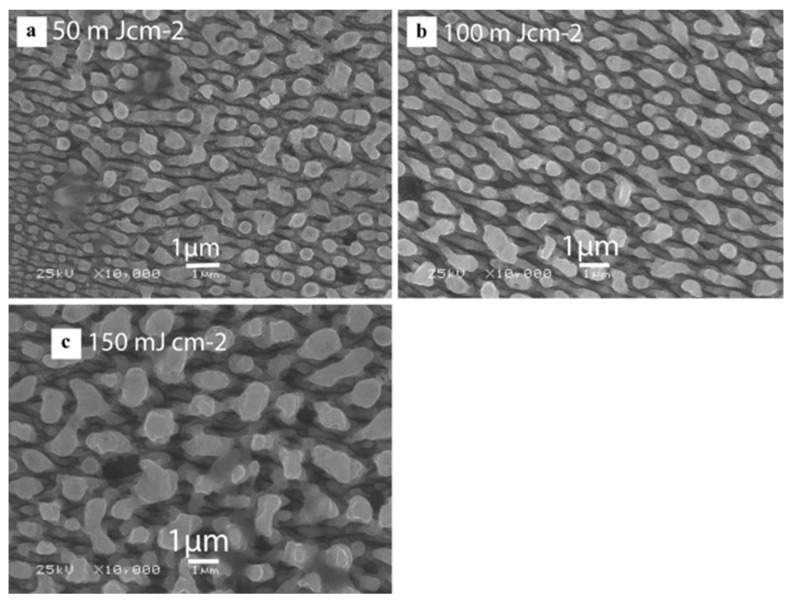
Dumbbell-shaped structure in ethanol environment. (**a**) 50 mJ cm^−2^; (**b**) 100 mJ cm^−2^; (**c**) 150 mJ cm^−2^. “Reprinted with permission from Ref. [38], Copyright (2013), Springer Nature”.

**Figure 11:**
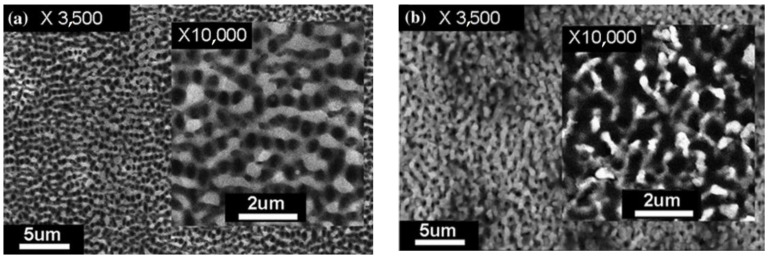
Porous structures in ethanol environment. (**a**) Quasiordered array of holes when the scan speed was 320 μm/s; (**b**) unordered netlike structure when the scan speed was 20 μm/s. “Reprinted with permission from Ref. [44], Copyright (2013), Elsevier”.

Bashir S. et al. [38] prepared the dumbbell-shaped structure shown in Figure 10 in an ethanol environment, and the size of irregular nanoparticles increased with the femtosecond laser’s fluence. Bian H. et al. [44] produced a microscale porous netlike structure and quasiordered holes of 280 to 320 nm on a stainless steel surface, as shown in Figure 11. For faster-speed (320 m/s) scanning, due to the bubble production and nonlinear optical effect [62], a gratinglike structure of the nanopores was obtained due to the disorderly energy distribution. When the scanning speed was reduced to 80 μm/s, the melting of the metal in the holes was enhanced, and the metal was pushed out under the mechanical force and resolidified by the surrounding liquid to form an island-protrusion structure. When the scanning speed was reduced to 20 μm/s, the porous netlike structure was finally formed due to the repeated melting and resolidification processes of the materials under the action of hydrodynamics.

**Figure 12:**
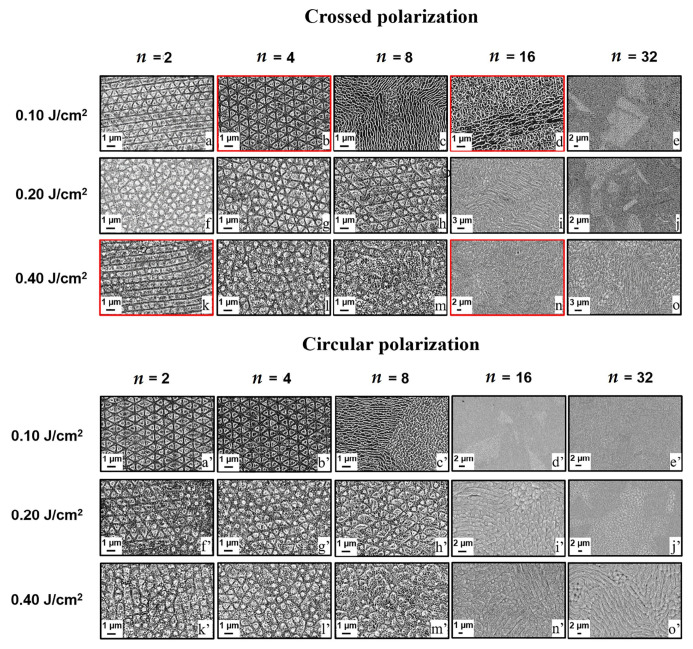
Triangular, pillarlike, and bushlike structures on AISI 301 with different polarization, fluence and number of sub-pulses. “Reprinted with permission from Ref. [34], Copyright (2019), Elsevier”.

Giannuzzi G. et al. [34] prepared triangular, pillarlike, and bushlike structures via application of cross-polarized and circular polarized bursts of femtosecond laser pulses on stainless steel surfaces, as shown in Figure 12. Table 2 provides a summary of main test parameters and typical structures of others.

## 3. Micro/Nano Periodic Surface Structure Performance

Compared with ordinary material surfaces, femtosecond-laser-induced micro/nano periodic surface structures exhibit different physical and chemical properties, and have great application potential in many aspects. For example, to improve the wetting performance, optical properties, and even multifunctional integrated properties of a material, they can be used for self-cleaning technology, liquid transportation without loss, reducing surface friction, color marking, and preparing grating structures. In addition, they can improve the surface Raman enhancement properties of a material, and can be used for optical signal detection.

### 3.1. Wetting Performance

**Figure 13:**
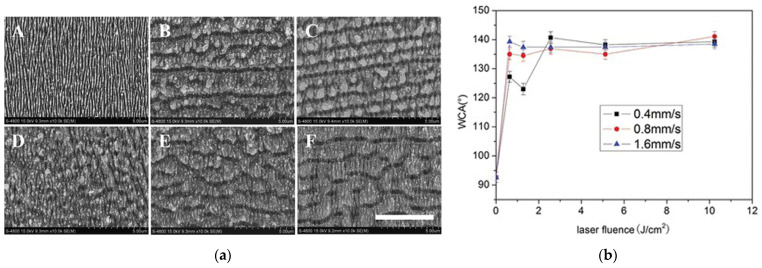
Surface structure and wetting performance of 316L [63]. (**a**) Micropatterns of the stainless steel surfaces induced by femtosecond laser with different laser fluence and scanning speeds in deionized water (scale bar is 5 µm); (**A**) 0.64 J/cm^2^, 0.4 mm/s; (**B**) 0.64 J/cm^2^, 0.8 mm/s; (**C**) 0.64 J/cm^2^, 1.6 mm/s; (**D**) 1.28 J/cm^2^, 0.4 mm/s; (**E**) 2.56 J/cm^2^, 0.4 mm/s; (**F**) 5.12 J/cm^2^, 0.4 mm/s; (**b**) effect of depth on the contact angles. Reprinted by permission of the publisher (Taylor & Francis Ltd., http://www.tandfonline.com, accessed on 23 January 2022).

Wetting performance is a very important property of the surface of solid materials, and also is the focus of the femtosecond laser micro/nano processing field of alloy structural materials. Extensive theoretical and experimental studies have shown that it mainly depends on the microgeometry of a material’s surface and the chemical composition and properties of the material’s surface. Chunyong Liang et al. [63] prepared hydrophobic and oleophilic ripples, grooves, and pores in a deionized water environment, as shown in Figure 13. The surface roughness increased with an increasing laser fluence while the corresponding water contact angle was increased (maximum 142.5°) and ethylene glycol contact angle was decreased (minimum 6.4°).

**Figure 14:**
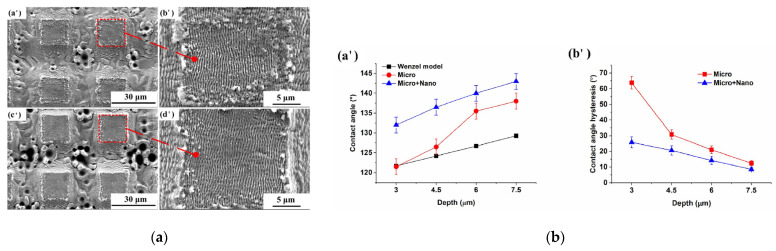
Surface structure and wetting performance of AISI 304 [55]. (**a**) Hierarchical structures at overscan number of (**a’**,**b’**) 9, (**c’**,**d’**) 15; (**b**) effect of depth on the contact angles of CAs (**a’**) and CAHs (**b’**). (Open Access).

Xingsheng Wang et al. [55] prepared an ordered hierarchical structures with highly controllable dimensions on a stainless steel surface using a picosecond laser in two steps, as shown in Figure 14. The first step was to prepare large-scale ripple structure of about 450 nm, while the second step used a laser direct-writing method to create a micro squared structure with a 19 μm width, 3~7.5 μm depth, and 19 μm interval. The order of this nanostructure before the microstructure could effectively obtain micro/nano hierarchical structures with a uniform coverage of nanostructures and with excellent hydrophobic performance, with a static water-contact angle of 143.0°. Martinez-Calderon et al. [64], however, used the steps of a microstructure before the nanostructure and developed a micro/nano layered structure on the surface of stainless steel with a static contact angle exceeding 150°. Camilo Florian et al. [65] used a similar scanning scheme to study the surface structure of steel (16MnCr5), and studied the influence of experimental parameters such as raster scans, interline spacing, and polarization direction; replicated the micro/nanostructure of the steel surface to the polymer surface; and studied the wetting performance of the polymer replication surface.

**Figure 15:**
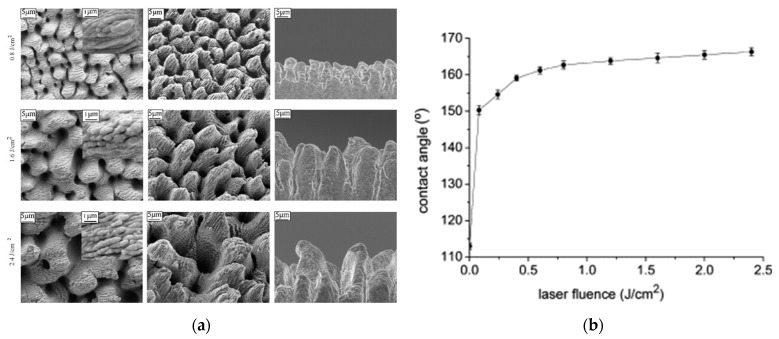
Surface structure and wetting performance of AISI 316L. (**a**) Micro/nano hierarchical structures; (**b**) apparent water CAs as a function of corresponding laser fluences. “Reprinted with permission from Ref. [56], Copyright (2009), Elsevier”.

**Figure 16:**
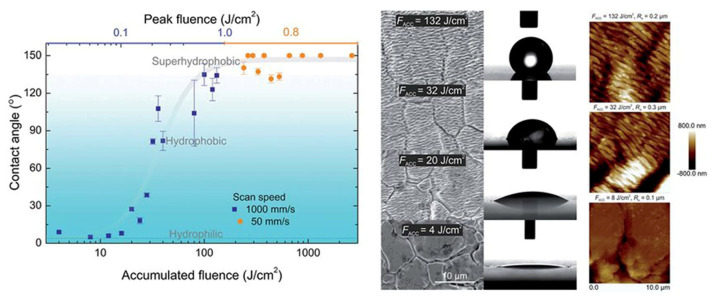
Surface structure and wetting performance of 1.4301 [68]. Available via license: Creative Commons Attribution-NonCommercial 3.0 Unported.

Bo Wu et al. [56] created superhydrophobic structures on a stainless steel surface using femtosecond laser irradiation and silanization, as shown in Figure 15. By increasing the femtosecond laser fluence, three micro/nanometer structures including an LIPSS structure covered by nanoparticles, a micron periodic ripple structure covered by LIPSS, and cone-shaped spikes covered by LIPSS were prepared successively. The conical hierarchical structure had the best hydrophobicity, with a maximum contact angle of 166.3° and a minimum slip angle of 4.2°, which highly agreed with the model analysis results. Sona Moradi [66] studied in detail the influence of femtosecond laser irradiation process parameters (laser fluence and scanning speed) on the micro/nanostructure morphology and its hydrophobicity on stainless steel, and obtained four typical structures (similar to those shown in Figure 16) by changing the test parameters. A wetting analysis showed that most of these structures were superhydrophobic, especially those with triple roughness. Hu S. et al. [67] prepared three-level hierarchical surfaces with a macro–micro–nanostructure through a special design that also achieved an excellent performance.

Yao C. et al. [69] found that 304 stainless steel showed a better hydrophilic performance after femtosecond laser treatment, with a static water contact angle as low as 37°. Sepehr Razi et al. [70] studied the chemical composition and wettability of stainless steel surfaces fabricated using a nanosecond laser in air and water environments, and obtained similar results. A.K. Singh et al. [71] used a femtosecond laser to produce self-aligned microprotrusions with an ultrahigh density (~10^6^/cm^2^) on the surface of 304 stainless steel. The microstructure initially showed superhydrophilic behavior, but underwent an interesting transition after 50 days in the air to a highly hydrophobic structure with a static water contact angle of 144°. Giannuzzi G. et al. obtained similar results [34]; their samples showed superhydrophobic properties after 55 days of placement, with a static water contact angle reaching 160°. Andrius Žemaitis et al. [68] studied the stainless steel 1.4301 (German brand), and established a relationship of the surface periodic structure and its wetting performance with the cumulative laser fluence of a femtosecond laser (see Figure 16). It was found that with an increase in the femtosecond-laser-accumulated laser fluence, the stainless steel surface gradually obtained a uniform ripple and spike structure, and the stable wetting performance also gradually changed from superhydrophilic to superhydrophobic.

The contact angles of a liquid and a material surface are usually calculated using the Wenzel [72] and Cassie [73] models. The Wenzel model assumes complete contact between the liquid and the material surface, with the actual contact angle related to the surface roughness and the contact angle of the liquid on a smooth surface. The Cassie model is based on the incomplete contact of the liquid and the material surface, and the actual contact angle is related to the proportion of air between the contact surfaces. It was found that when the micro- and nanostructures were more complex and the ablation depth was deeper, more air could gather in the micro and nanostructure space, and the liquid could not completely wet the entire surface. In this case, the Cassie model and experimental results showed better agreement [55,56].

### 3.2. Other Performances

Caizhen Yao et al. [69] studied the optical properties of stainless steel micro/nanostructures, and found that they could significantly reduce the light reflection, which was due to the scattering and absorption of the micro/nanostructures.

Wu B. et al. [56] studied the corrosion resistance of stainless steel in H_2_SO_4_ and NaCl solutions after femtosecond laser treatment, and found that the corrosion resistance became worse, but improved with the improved hydrophobic performance of the surface structure. However, Trdan U. et al. [74] confirmed a perfect correlation between the wettability and corrosion resistance of micro and nano surface structures, obtaining a superhydrophobic surface with a contact angle of 168 ± 3.0°, and resulting in an enhanced corrosion resistance.

Zhuo Wang [75,76] studied the friction performance of stainless steel after femtosecond laser treatment, and found that the preparation of a micron groove and nanoripple structure could effectively reduce friction and reduce wear. The micro/nanostructure on the one hand could store lubricant and was conducive to lubrication, while on the other hand, it could store particles resulting from wear to further reduce friction. Lijun Yang et al. [77] prepared four micro/nanostructures with different shapes and studied their friction properties. The results of unidirectional rotating friction tests showed that a spherical structure with a depth-to-width ratio of 0.2 could significantly improve the tribological performance under low loading and speed conditions. Gnilitskyi I. et al. found that a micro/nano surface structure prepared using high-speed scanning reduced wear by 65% [78].

Table 3 provides a summary of typical surface structures and performances of the test materials and the main test parameters used for the fabrication of micro/nano surface structures.

## 4. Several Laws and Challenges Revealed by the Research

### 4.1. Micro/Nano Periodic Structure Evolution

The formation process of a femtosecond-laser-induced surface ripple structure is a complex nonlinear, nonequilibrium process that involves many optical, physical, chemical, and mechanical principles. The induced micro/nano periodic structure changes regularly with the parameters of femtosecond laser experiments. Existing research [35] revealed the transition of nano HSFLs (∥) to nano LSFLs (⊥), and other research [36] also revealed the transition of nano LSFLs (⊥) to a micron ripple/groove (∥). In addition, there are also research findings regarding the transformation of nano LSFLs (⊥) to nano HSFLs (⊥) [49,50]. Combined with the existing research results, there are indications that the transition of nano HSFLs (∥) to nano LSFLs (⊥) and then to a micron ripple/groove (∥) is a continuous transition process. Therefore, a systematic and in-depth study is needed to reveal the orderly change law of the period and direction of micro/nanoripples.

For the formation of micro/nano periodic structures, interference [79,80,81], self-organization [12,82], second harmonic generation [13,83], surface plasma [84,85], and Coulomb explosion [86] have been successively proposed. However, these theoretical models can only be applied under specific experimental conditions, so the formation mechanism of the ripple structure requires further investigation.

### 4.2. Effects of the Femtosecond Laser Parameters

**Figure 17:**
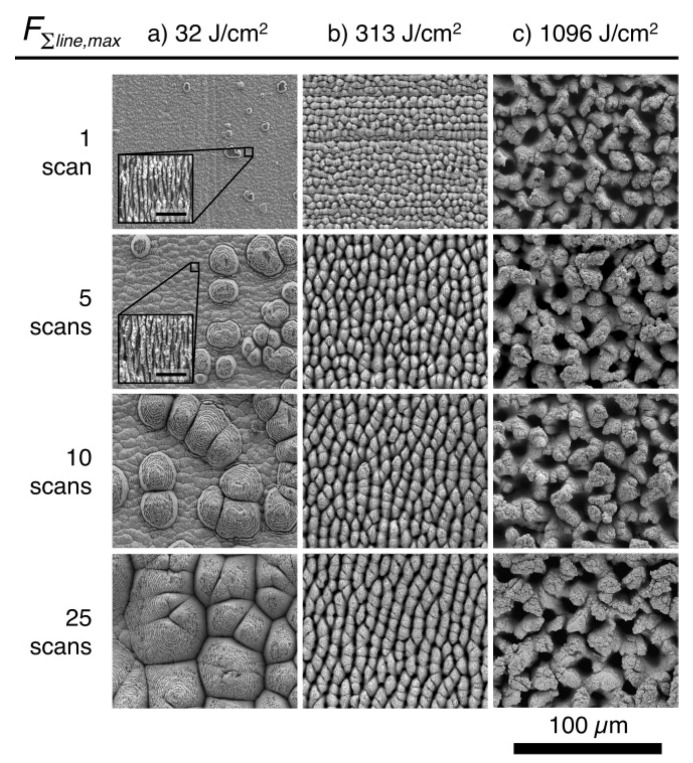
Evolution of micro/nano periodic surface structure with cumulative energy densities and scan times. (**a**) Ellipsoidal cones; (**b**) Columnar structures; (**c**) Chaotic structures. “Reprinted with permission from Ref. [60], Copyright (2015), Elsevier”.

There are many femtosecond laser parameters that affect the micro/nano surface structure, and many factors are not independent, as laser fluence, repetition frequency, number of pulses, and scanning speed will affect the energy received per unit area per unit time. Ling E. J. Y. et al. [60] made beneficial attempts to integrate the power density, scan speed, distance from the focal point, and line overlap into one parameter to obtain the evolution law of micro/nano periodic surface structures under different cumulative energy densities and scan times, as shown in Figure 17.

In order to define a more comprehensive and accurate evolution law and obtain a micro/nano periodic structure with good performance and application prospects, it is necessary to carry out systematic in-depth research and to establish a mathematical model between experimental parameters and periodic structures, and to seek the optimal solution. For specific application materials, a large number of basic and systematic experiments need to be carried out to establish the changing map of surface micro/nano periodic structure, and then guide the subsequent production and application.

Meanwhile, the processing speed of femtosecond laser micro/nanomachining is also a key factor that restricts the application of femtosecond lasers. Most studies use a processing speed at the mm/s level, and some studies have confirmed [65,78] that using an m/s-level fast processing speed can also obtain a satisfactory organization and performance that can meet the application’s needs. This type of fast machining process still requires more research in order to verify and perfect it.

### 4.3. Influence of Materials’ Condition and Microstructure

**Figure 18:**
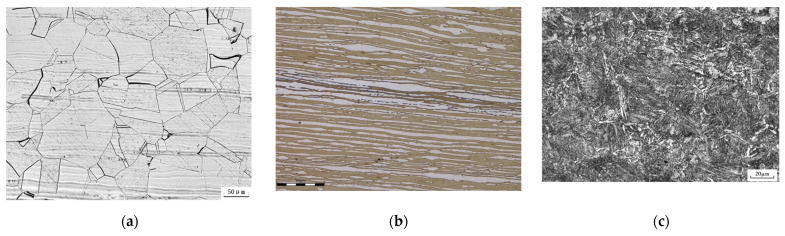
Typical microstructures of materials used in the oil and gas industry: (**a**) austenite stainless steel; (**b**) duplex stainless steel; (**c**) N80Q casing.

The surface conditions and microstructures of metal structural materials have important effects on the preparation and study of micro/nano periodic surface structures. To study the generation and evolution of micro/nano periodic surface structures, samples are usually polished to the nanoscale. However, the physical surface of the metal structural material has a high degree of roughness, which poses a severe challenge to the application of femtosecond laser micro/nano processing technology to the physical surface. At the same time, at present, the preparation of a femtosecond-laser-induced surface micro/nano periodic structure mainly uses a pure metal or alloy with a single microstructure, but the metal structural material is usually more complex (as shown in Figure 18b,c), so whether a uniform micro/nano periodic structure can be obtained needs to be studied systematically.

## 5. Conclusions

Functional micro/nanostructures have become an important direction in global science and technology development because they show different material and functional characteristics than at the macroscale. Researchers used femtosecond lasers to carry out systematic and in-depth research on metal structural materials (mainly stainless steel), resulting in a large body of useful scientific research for reference.

(1) The shape, size, and period of the micro/nano periodic structures produced on the surface of femtosecond laser ablation materials are not only closely related to the properties of the material itself, but also depend on the experimental research environment and the laser parameters used. Surface structures with different morphology and periods have been prepared under different experimental environments and laser-processing parameters. The researchers focused on the laser fluence, pulse number, scanning speed, bursts of pulses, and processing environment, and gained some regularity of understanding.
(2) In a lower laser fluence condition, the nanoscale LIPSS structure and the microscale groove structure can be obtained. The period and direction of the ripple structure will change regularly with the laser parameters. The ablation threshold of nano HSFLs (∥) is the lowest, and with an increase in the laser fluence, will gradually change to nano LSFLs (⊥) and then to microripples/grooves (∥). At a higher laser fluence, micro and nano hierarchical structures can be obtained, such as the moth-eye structure, porous mesh structure, mound/conical structure, and columnar structure.
(3) The ripple structures formed in an air environment are relatively continuous and neat, while the ripple structures formed in a liquid environment such as deionized water and ethanol become discontinuous due to the uneven energy distribution and hydrodynamic effects, and are cut into short strips and dumbbell structures. Cross-polarization and circular-polarization femtosecond lasers are used to prepare a more complicated and changeable structure.
(4) Compared with an ordinary material surface, the femtosecond-laser-induced micro/nano periodic surface structure shows different physical and chemical properties. Focusing on wetting performance, corrosion performance, and friction properties, the research confirmed that micro/nano multiple-roughness hierarchical structures showed excellent properties and have a bright application prospect. However, there are still some challenges in practical engineering applications. For example, the surface state (roughness) and microstructure (crystal boundary distribution) of metal structural materials have an important impact on the preparation and performance of micro/nano periodic surface structures, and require further in-depth research and exploration.

## Figures and Tables

**Table 1:** Main test parameters and typical structures of ripples.

Material	Laser Wavelength λ nm	Pulse Width *t* fs ^a^	Repetition Frequency *f* kHz	Laser Fluence *F* J/cm^2^	Processing Environment	Period of Ripples Λ nm ^b^	Direction	Reference
AISI 420 AISI 304	780	164	1	1.32	Air	526	^c^	[35]
AISI 420 AISI 304	780	164	1	0.67	Air	310	^c^	[35]
X40Cr14	800	125	5	0.10~1.66	Air	500~680	⊥	[36]
X40Cr14	800	125	5	1.51~4.98	Air	1.6~4.2 μm	∥	[36]
304	800	35	0.004~1	0.70	Air	480~620	⊥	[32]
304	800	35	0.004~1	0.70	Air	1.4~2.3 μm	∥	[32]
SS304	800	35~260	0.1	0.15~0.37	Air	540~700	^c^	[47]
AISI 52100	790	130	1	0.32	Air	590~630	^c^	[39]
STAVAX	800	185	1	0.21	Air	400	⊥	[37]
AISI 443	800	120	1	1.33	Air, water, and methanol	<1 μm	⊥	[40]
AISI 304	800	30	1	0.55	Air	550	^c^	[44]
AISI 304	800	30	1	0.55	Ethanol	270~340	⊥	[44]
AISI 304	800	25	1	0.05~0.15	Air	400~500	^c^	[38]
AISI 304	800	25	1	0.05~0.15	Air	200~250	^c^	[38]
AISI 304	800	25	1	0.05~0.15	Water	200~400	^c^	[38]
AISI 304	800	25	1	0.05~0.15	Ethanol	200~400	^c^	[38]
AISI 304	532	10 ps	10	0.25~1.44	Air	450	⊥	[51]
AISI 304	532	10 ps	10	0.25~1.44	Air	100~130	∥	[51]
AISI 304	532	10 ps	10	0.54~1.44	Air	1.2 μm	∥	[51]
A stainless steel	1064	10 ps	1	0.27	Air	100~200	∥	[50]
A stainless steel	1064	10 ps	1	0.27	Air	800	⊥	[50]
A stainless steel	1064	10 ps	1	0.27	Air	250~400	⊥	[50]
A stainless steel	800	50	0.001~1	0.07~0.18	Air	400~600	⊥	[49]
A stainless steel	800	50	0.001~1	0.07~0.13	Air	270~310	⊥	[49]
316L	800	100	1	0.41~0.52	Air	530~720	⊥	[41]
AISI 304	532	10 ps	50	0.32	Air	450	⊥	[55]
AISI 316L	800	130	1	0.08~0.20	Vacuum	500	⊥	[56]
Nitrided 304	800	180	1	0.08~0.22	Air	250~670	⊥	[48]
316L	257	350	250	0.11	Air	76 ± 2	∥	[43]
316L	257	350	250	0.11	Air	153 ± 4	⊥	[43]
316L	257	350	250	0.11	Air	426 ± 7	∥	[43]

Note: ^a^ fs, except as otherwise marked; ^b^ nm, except as otherwise marked; ^c^ not reported in the text.

**Table 2:** Main test parameters and typical structures of others.

Material	Laser Wavelength λ nm	Pulse Width *t* fs	Repetition Frequency *f* kHz	Laser Fluence *F* J/cm^2^	Processing Environment	Typical Structures	Reference
X40Cr14	800	125	5	2.49~	Air	Porous structure Hole size: 1~4 μm	[36]
X40Cr14	800	125	5	4.15~	Air	Splashlike structure	[36]
304	800	35	0.004~1	0.7	Air	Moth-eye-like structure Hole size: 1.7~5 μm	[32]
AISI 443	800	120	1	1.33	Air	N-mounds/cones	[40]
AISI 443	800	120	1	1.33	Methanol	Ripple-textured microprotrusions	[40]
AISI 304	800	30	1	0.55	Water	Ripple-covered random microstructures	[44]
AISI 304	800	30	1	0.55	Ethanol	Gratinglike structure Hole size: 280~320 nm	[44]
AISI 304	800	30	1	0.55	Ethanol	Porous netlike structure Nanorods: 100~200 nm × 1~2 μm	[44]
AISI 304	800	25	1	0.05~0.15	Ethanol	Dumbbell-shaped structure	[38]
AISI 443	800	120	1	0.70~1.66	Air	*N*-mounds/cones	[59]
304	800	<100	10	313 ^a^	Air	Columnar structure	[60]
304	800	<100	10	1096 ^a^	Air	Chaotic structure	[60]
AISI 301	1030	200	200	0.1~0.4	Air	Triangular bushlike structures	[34]

Note: ^a^ cumulative line laser fluence.

**Table 3:** Surface structure and performances of test materials.

Material	Laser Wavelength λ nm	Pulse Width *t* fs	Repetition Frequency *f* kHz	Laser Fluence *F* J/cm^2^	Scan Speed *υ* mm/s	Processing Environment	Surface Structure	Main Property	Reference
316L	800	50	1	0.64~10.24	0.4~1.6	Water	Ripples, holes	Hydrophobic and oleophilic	[63]
AISI 304	532	10 ps	50	0.32, 5.1	40	Air	Ripples, micromatrix	Hydrophobic	[55]
AISI 316L	800	130	1	0.08~2.4	1	Vacuum	Ripples, cones	Superhydrophobic	[56]
304	800	35	0.1	0.3~1.0	0.36	Air	Ripples	Hydrophilic	[69]
304SS	800	50	3	0.9	0.4	Air	Microprotrusions	Hydrophilic→ hydrophobic	[71]
1.4301	1026	170	100	0.08~0.84	1~50	Air	Ripples, spikes	Hydrophilic→ Superhydrophobic	[68]
AISI 304	800	130	1	25.2	1	Air	Ripples, micromatrix	Hydrophilic→ superhydrophobic	[64]
316L	800	150	1	1.5~480	0.25~1.85	Air	Ripples, columnar protrusions, cones	Superhydrophobic	[66]
AISI 316L	1064	40ns	25	62.28	150	Air	Micro grating structure	Superhydrophobic, corrosion-resistant	[74]
AISI 304L	800	130	1	>0.1	/	Air	Microgrooves	Wear-resistant	[75]
AISI 304L	800	130	1	0.36	0.2	Air	Nanoripples	Wear-resistant	[76]
GCr15	1030	255	50~200	>0.52	1~2	Air	Circle, triangle, square, rhombus	Wear-resistant	[77]
X5CrNi1810	1030	213	600	0.51	3000	Air	Ripples	Wear-resistant	[78]
